# Supplementary material for: Macrolactin XY, a Macrolactin Antibiotic from Marine-Derived Bacillus subtilis sp. 18
Source: Mar Drugs. 2024 Jul 23;22(8):331. doi: 10.3390/md22080331 (PMC11355411; doi:10.3390/md22080331)

Supplementary data

Macrolactin XY, a Macrolactin Antibiotic from Marine-Derived *Bacillus subtilis* sp.18

Figure S1. UV spectrum of macrolactin XY (**1**).
Figure S2. ^{1}H NMR of macrolactin XY (**1**) in $CDCl_3$.
Figure S3. ^{13}C NMR of macrolactin XY (**1**) in $CDCl_3$.
Figure S4. DEPT135 of macrolactin XY (**1**) in $CDCl_3$.
Figure S5. COSY of macrolactin XY (**1**) in $CDCl_3$.
Figure S6. HSQC of macrolactin XY (**1**) in $CDCl_3$.
Figure S7. HMBC of macrolactin XY (**1**) in $CDCl_3$.
Figure S8. NOESY of macrolactin XY (**1**) in $CDCl_3$.
Figure S9. HR-ESI-MS of macrolactin XY (**1**).
Figure S10. ORD of macrolactin XY (**1**).
Figure S11. UV spectrum of (5*R*, 9*S*, 10*S*)-5-(hydroxymethyl)-1,3,7-decatriene-9,10-diol (**2**).
Figure S12. ^{1}H NMR of (5*R*, 9*S*, 10*S*)-5-(hydroxymethyl)-1,3,7-decatriene-9,10-diol (**2**) in CD_3OD.
Figure S13. ^{13}C NMR of (5*R*, 9*S*, 10*S*)-5-(hydroxymethyl)-1,3,7-decatriene-9,10-diol (**2**) in CD_3OD.
Figure S14. DEPT135 of (5*R*, 9*S*, 10*S*)-5-(hydroxymethyl)-1,3,7-decatriene-9,10-diol (**2**) in CD_3OD.
Figure S15. COSY of (5*R*, 9*S*, 10*S*)-5-(hydroxymethyl)-1,3,7-decatriene-9,10-diol (**2**) in CD_3OD.
Figure S16. HSQC of (5*R*, 9*S*, 10*S*)-5-(hydroxymethyl)-1,3,7-decatriene-9,10-diol (**2**) in CD_3OD.
Figure S17. HMBC of (5*R*, 9*S*, 10*S*)-5-(hydroxymethyl)-1,3,7-decatriene-9,10-diol (**2**) in CD_3OD.
Figure S18. NOESY of (5*R*, 9*S*, 10*S*)-5-(hydroxymethyl)-1,3,7-decatriene-9,10-diol (**2**) in CD_3OD.
Figure S19. HR-ESI-MS of (5*R*, 9*S*, 10*S*)-5-(hydroxymethyl)-1,3,7-decatriene-9,10-diol (**2**).
Figure S20. ORD of (5*R*, 9*S*, 10*S*)-5-(hydroxymethyl)-1,3,7-decatriene-9,10-diol (**2**).
Figure S21. ECD of (5*R*, 9*S*, 10*S*)-5-(hydroxymethyl)-1,3,7-decatriene-9,10-diol (2) (Six additional isomers)

Figure S1. UV spectrum of macrolactin XY (**1**).

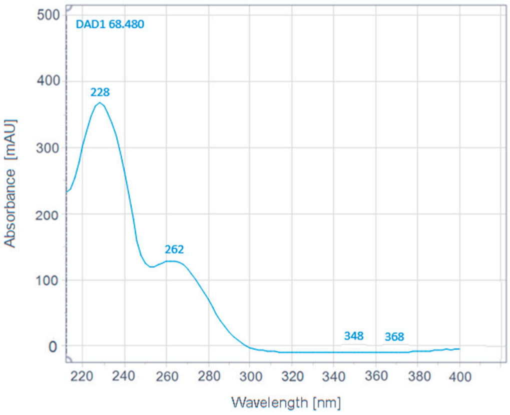

Figure S2. ^{1}H NMR of macrolactin XY (**1**) in $CDCl_3$.

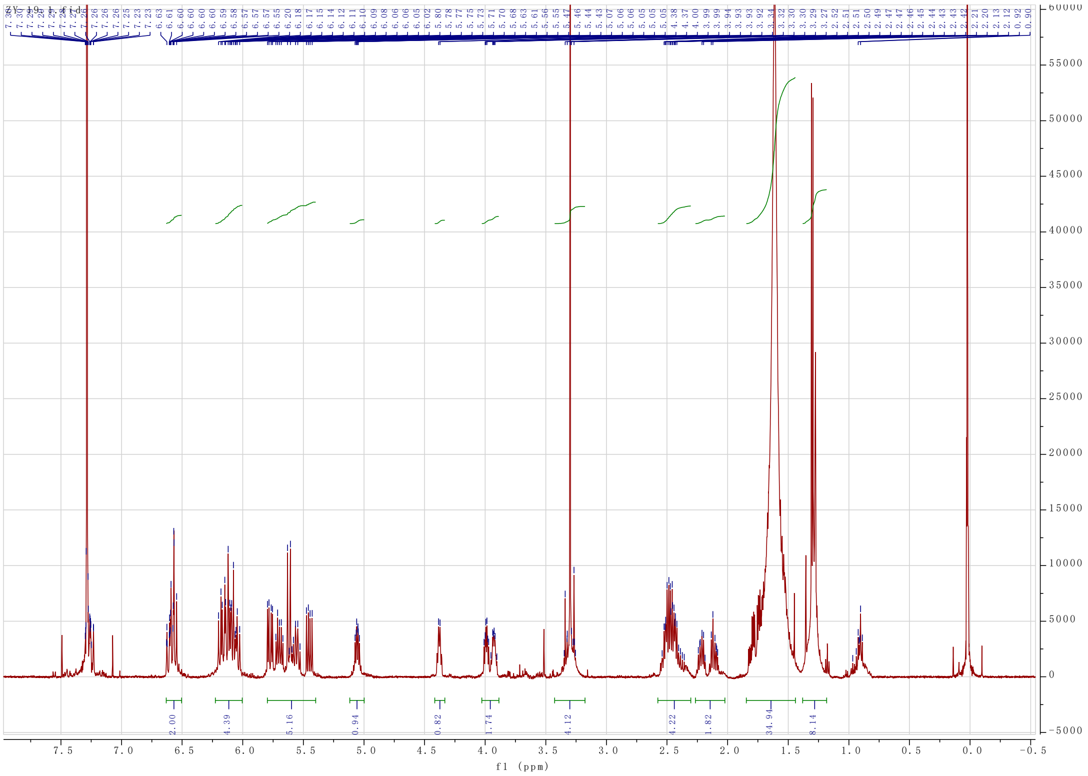

Figure S3. ^{13}C NMR of macrolactin XY (**1**) in $CDCl_3$.

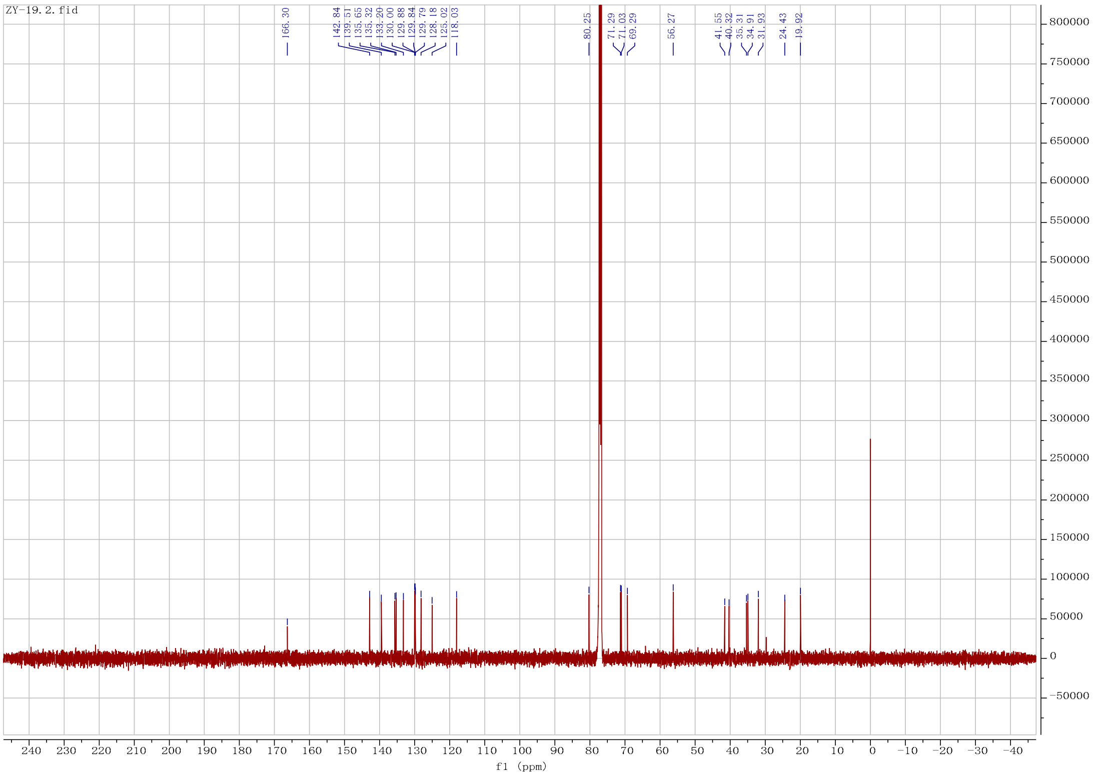

Figure S4. DEPT135 of macrolactin XY (**1**) in $CDCl_3$.

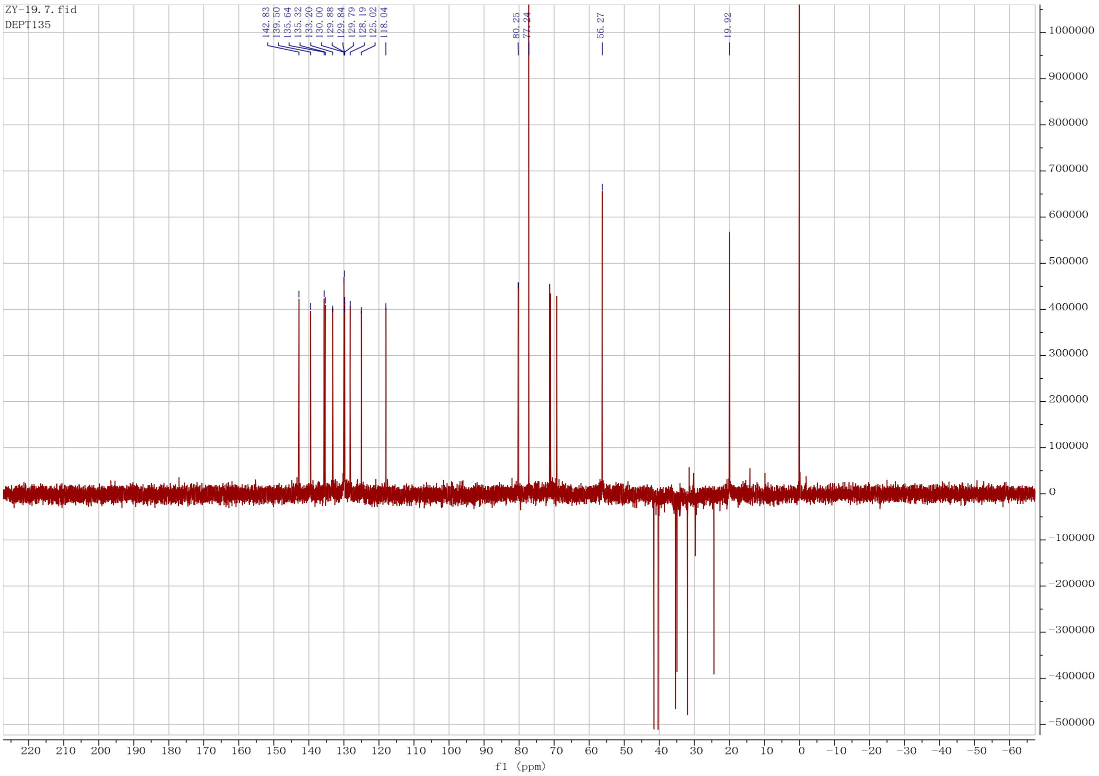

Figure S5. COSY of macrolactin XY (**1**) in $CDCl_3$.

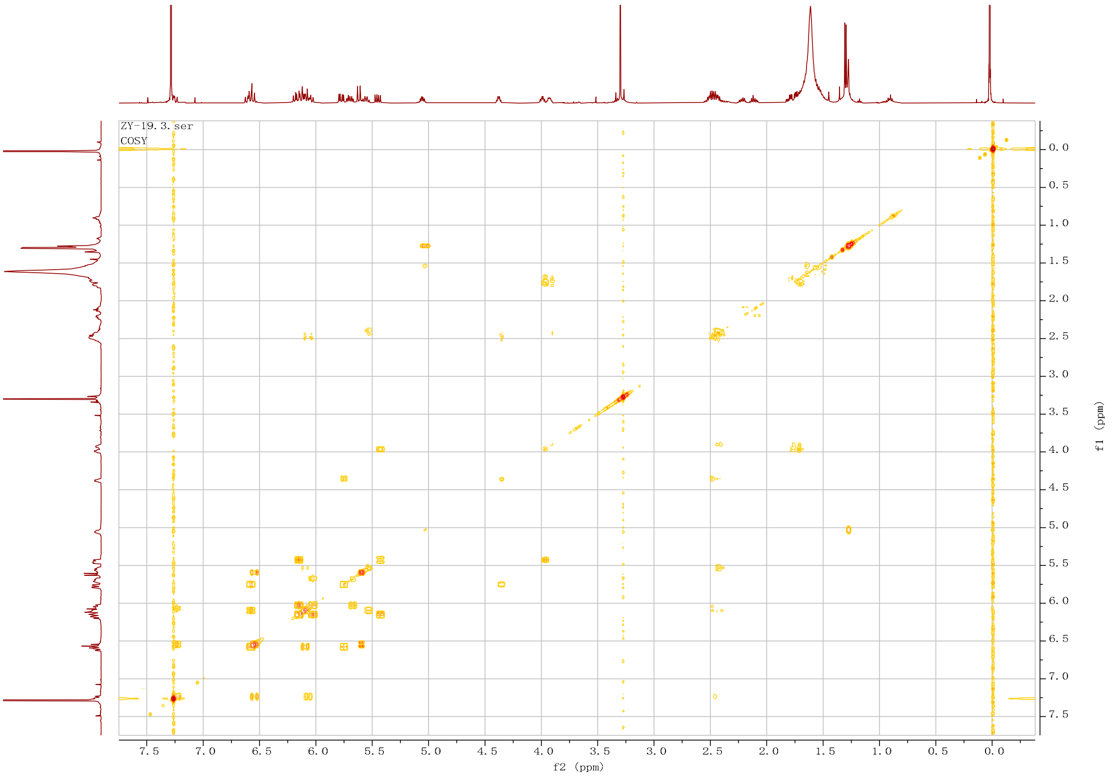

Figure S6. HSQC of macrolactin XY (**1**) in $CDCl_3$.

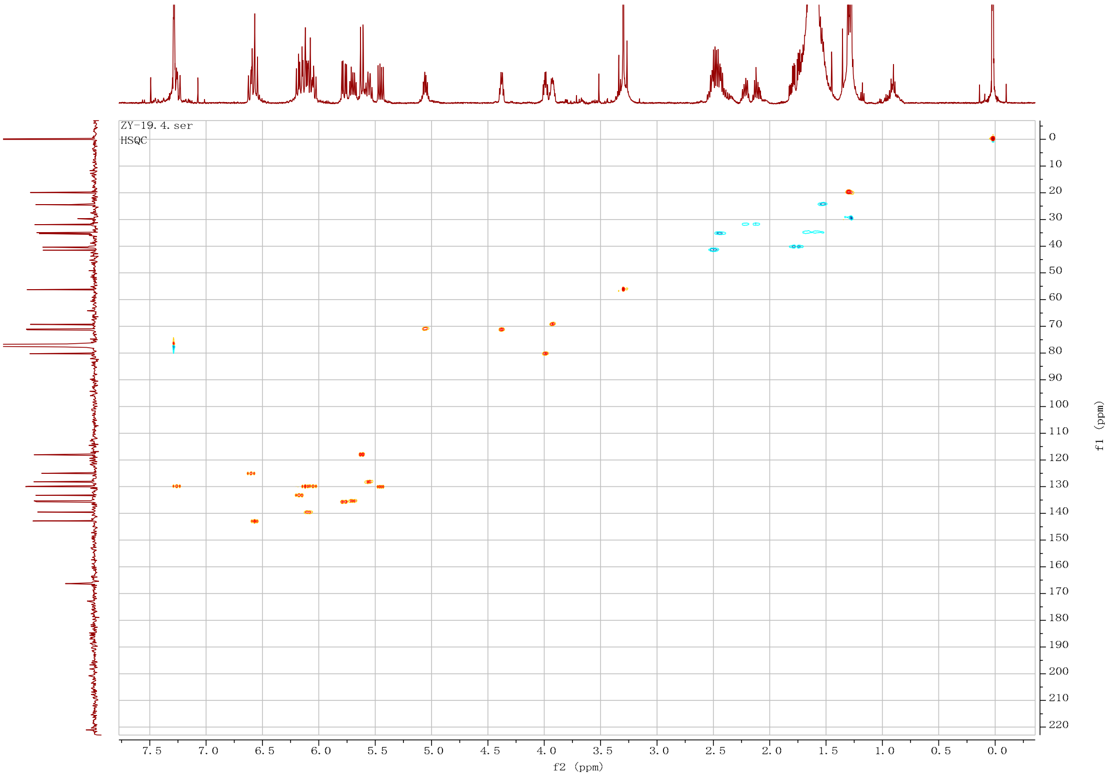

Figure S7. HMBC of macrolactin XY (**1**) in CDCl$_3$.

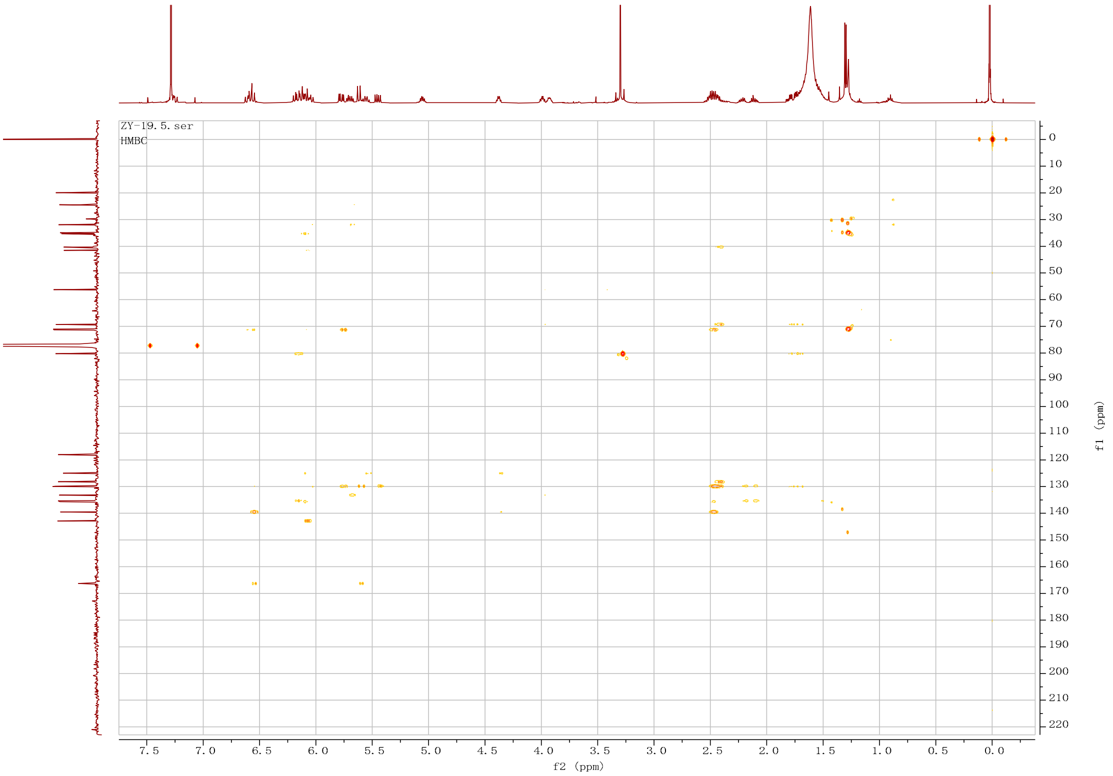

Figure S8. NOESY of macrolactin XY (**1**) in CDCl$_3$.

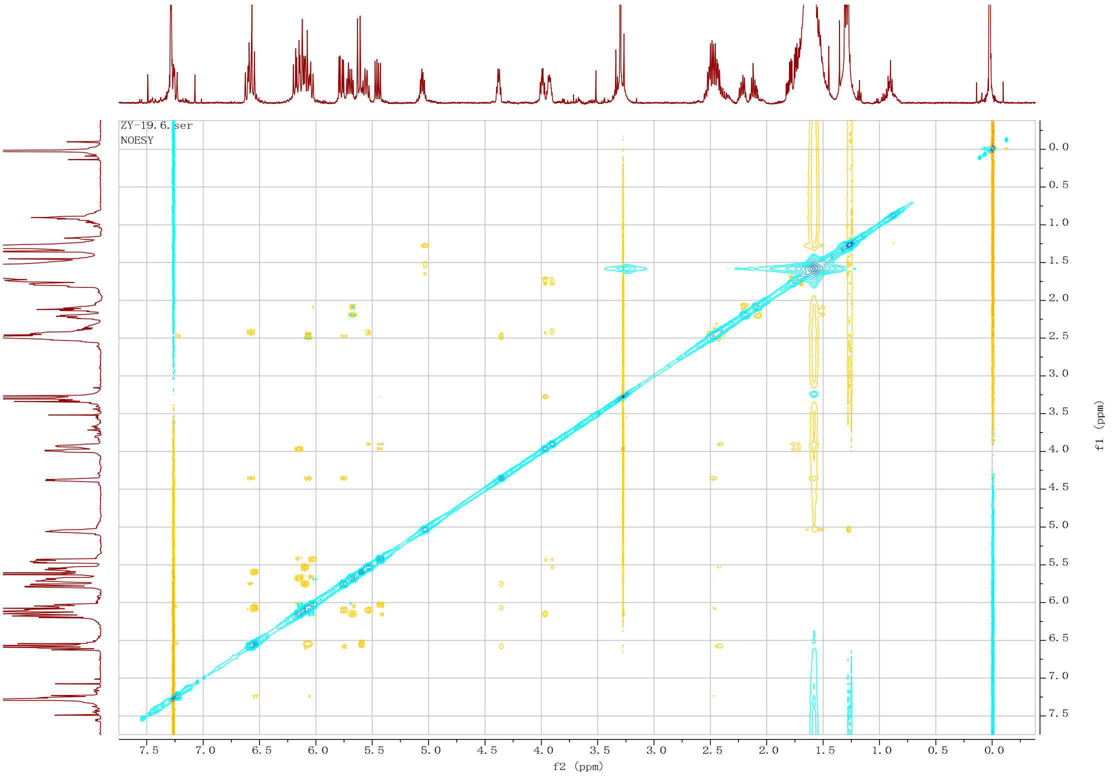

Figure S9. HR-ESI-MS of macrolactin XY (**1**).

Spectrum

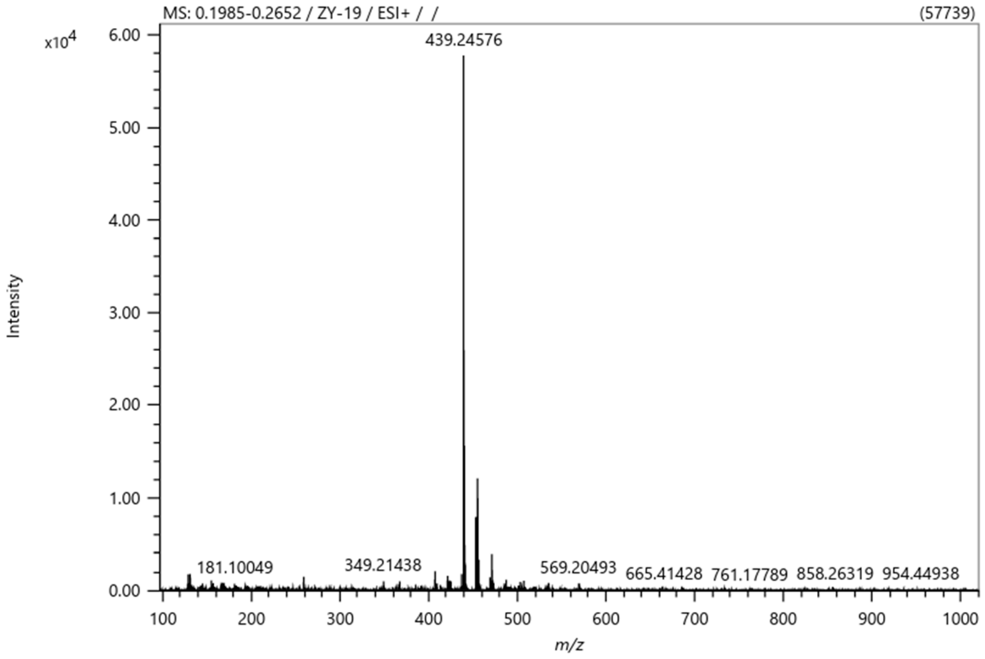

Elemental Composition

Parameters	
Tolerance:	±5.00 ppm
Electron:	Odd/Even
Charge:	+1
DBE:	-1.5 - 200.0

Elements Set 1:

Symbol	C	H	N	O	Na	S	Cl	Br
Min	0	0	0	0	1	0	0	0
Max	200	200	0	8	1	0	0	0

Symbol	Si	F	B
Min	0	0	0
Max	0	0	0

Results

Mass	Intensity	Intensity [%]	Formula	Calculated Mass	Mass Difference [mDa]	Mass Difference [ppm]	DBE
439.24576	57739.12	100.00	C25 H36 O5 Na	439.24550	0.26	0.59	7.5

Figure S10. ORD of macrolactin XY (**1**).

SIOC
Lingling Road 345
Shanghai
China

Anton Paar MCP 5500 - Measurement Results:

Software version: 4.00.11383.92
MCP serial number: 99030100

Sample Information:

- Unique Sample Id: 51602
- Date: 2023-1-10
- Time: 15:17:45
- Method: Specific Rotation (25°C)
- Master Condition: valid
- Sample Name: ZY-19
- Concentration: 0.0500 g/100cm³
- User: student

Measurement Result:

Sub Measurement Number	Unique Sample Id	Time	Optical Rotation	Sample Cell Temperature	Specific Rotation (calc.)
			[°]	[°C]	[°]
1	51603	15:16:27	-0.0274	25.04	-54.7945
2	51604	15:16:45	-0.0287	25.00	-57.3943
3	51605	15:17:04	-0.0297	24.99	-59.3941
4	51606	15:17:24	-0.0306	24.98	-61.1939
5	51607	15:17:43	-0.0313	24.98	-62.5937
average	51602	15:17:45	-0.0295	25.00	-59.0741
std. dev.			0.001381	0.0223	2.761168

GxP Information (at 589 nm):

- Last Quartz Adjustment: 2021-11-30 15:41:39 by Administrator

2023110 | 15:18:01

()

Figure S11. UV spectrum of (5*R*, 9*S*, 10*S*)-5-(hydroxymethyl)-1,3,7-decatriene-9,10-diol (**2**).

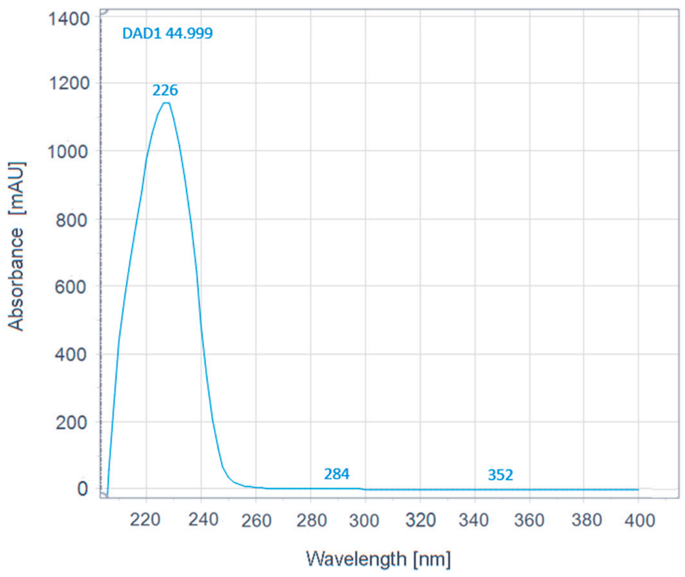

Figure S12. ^{1}H NMR of (5*R*, 9*S*, 10*S*)-5-(hydroxymethyl)-1,3,7-decatriene-9,10-diol (**2**) in CD_3OD.

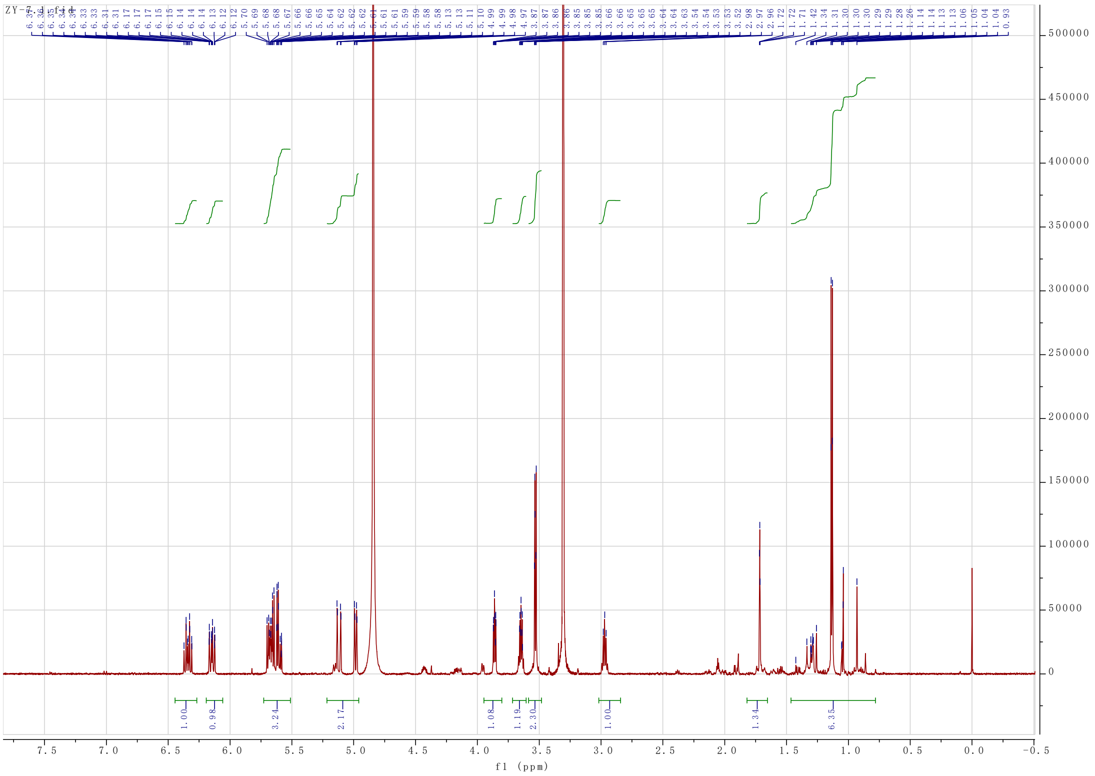

Figure S13. ^{13}C NMR of (5*R*, 9*S*, 10*S*)-5-(hydroxymethyl)-1,3,7-decatriene-9,10-diol (**2**) in CD_3OD.

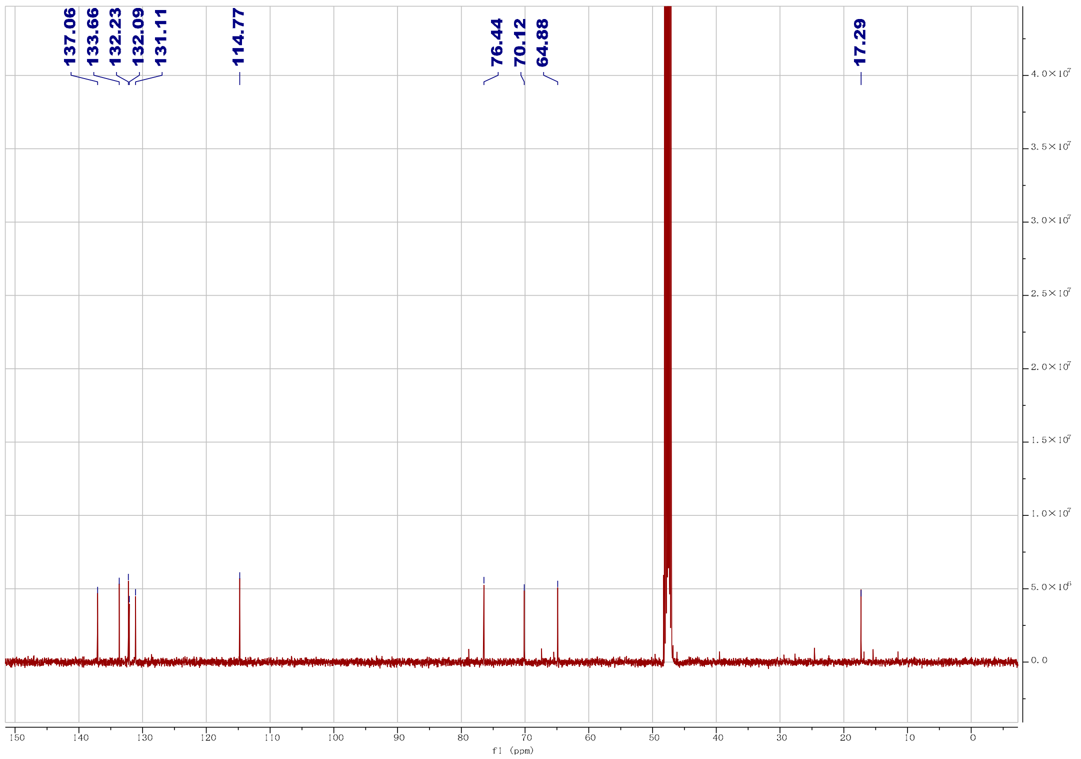

Figure S14. DEPT135 of (5*R*, 9*S*, 10*S*)-5-(hydroxymethyl)-1,3,7-decatriene-9,10-diol (**2**) in CD_3OD.

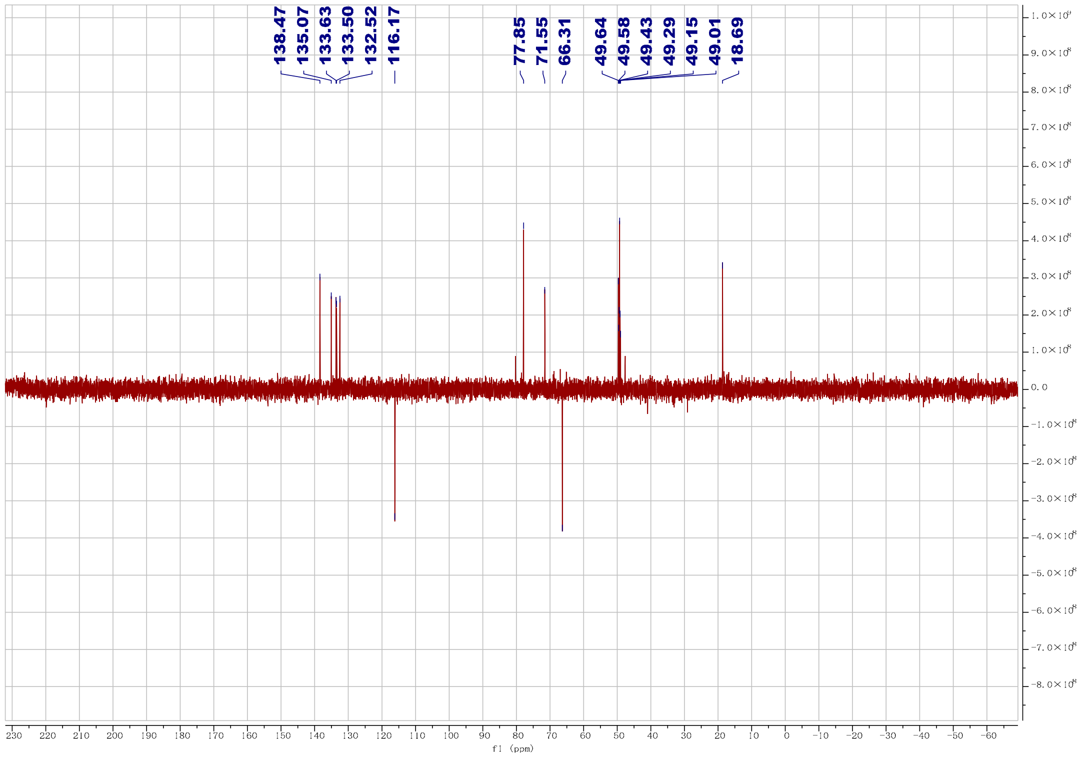

Figure S15. COSY of (5*R*, 9*S*, 10*S*)-5-(hydroxymethyl)-1,3,7-decatriene-9,10-diol (**2**) in CD_3OD.

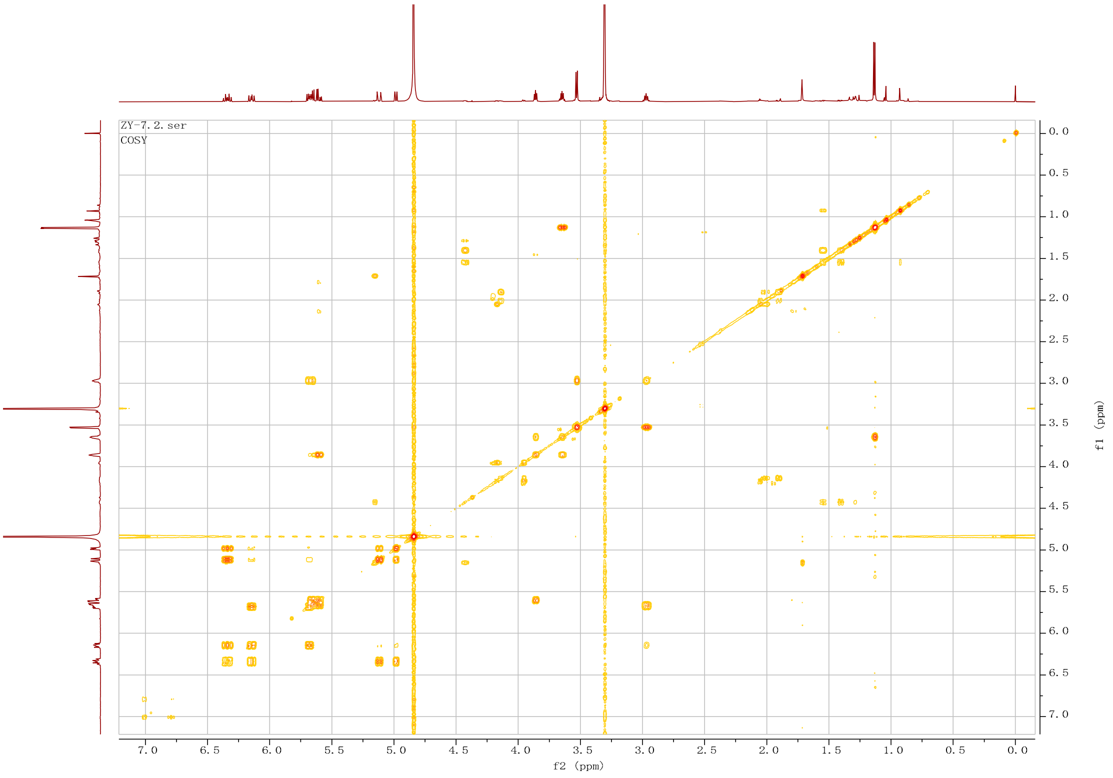

Figure S16. HSQC of (5*R*, 9*S*, 10*S*)-5-(hydroxymethyl)-1,3,7-decatriene-9,10-diol (**2**) in CD_3OD.

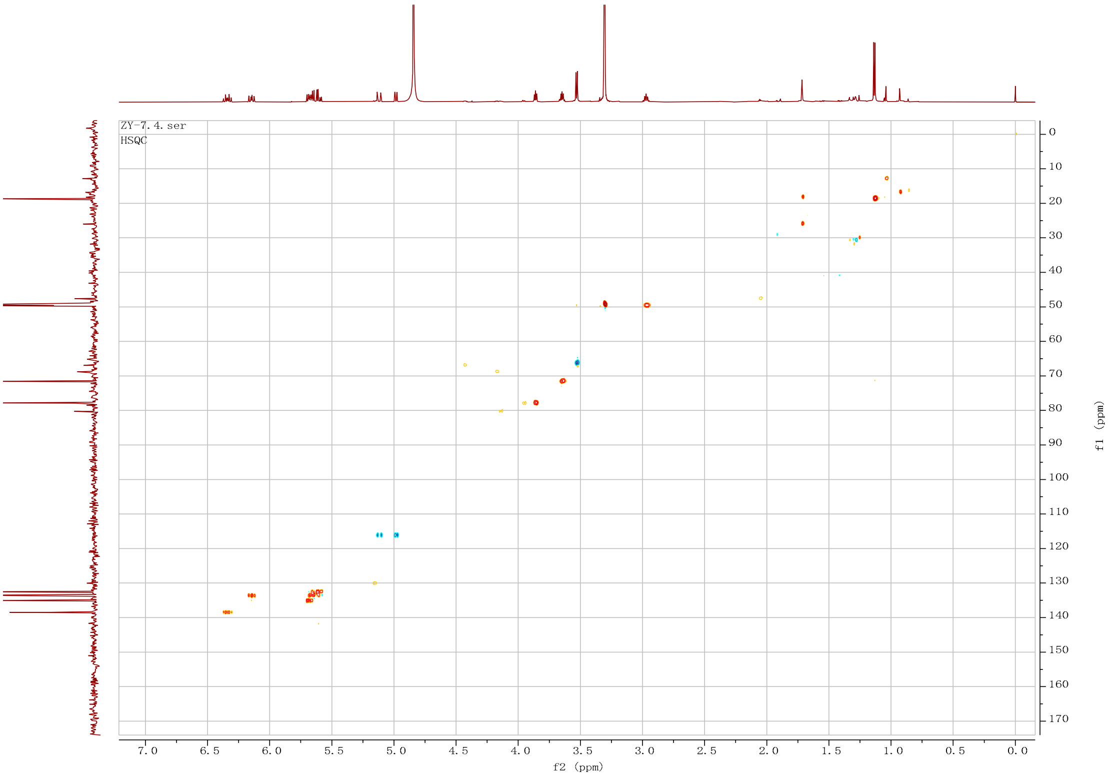

Figure S17. HMBC of (5*R*, 9*S*, 10*S*)-5-(hydroxymethyl)-1,3,7-decatriene-9,10-diol (**2**) in CD_3OD.

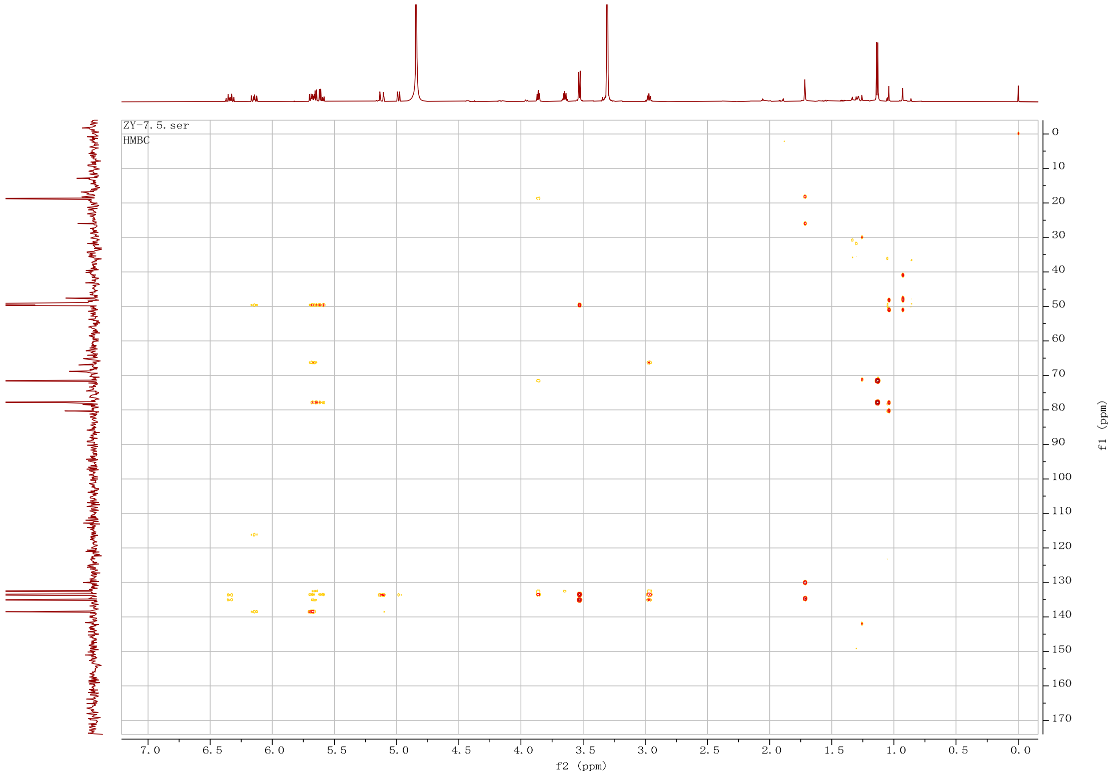

Figure S18. NOESY of (5*R*, 9*S*, 10*S*)-5-(hydroxymethyl)-1,3,7-decatriene-9,10-diol (**2**) in CD_3OD.

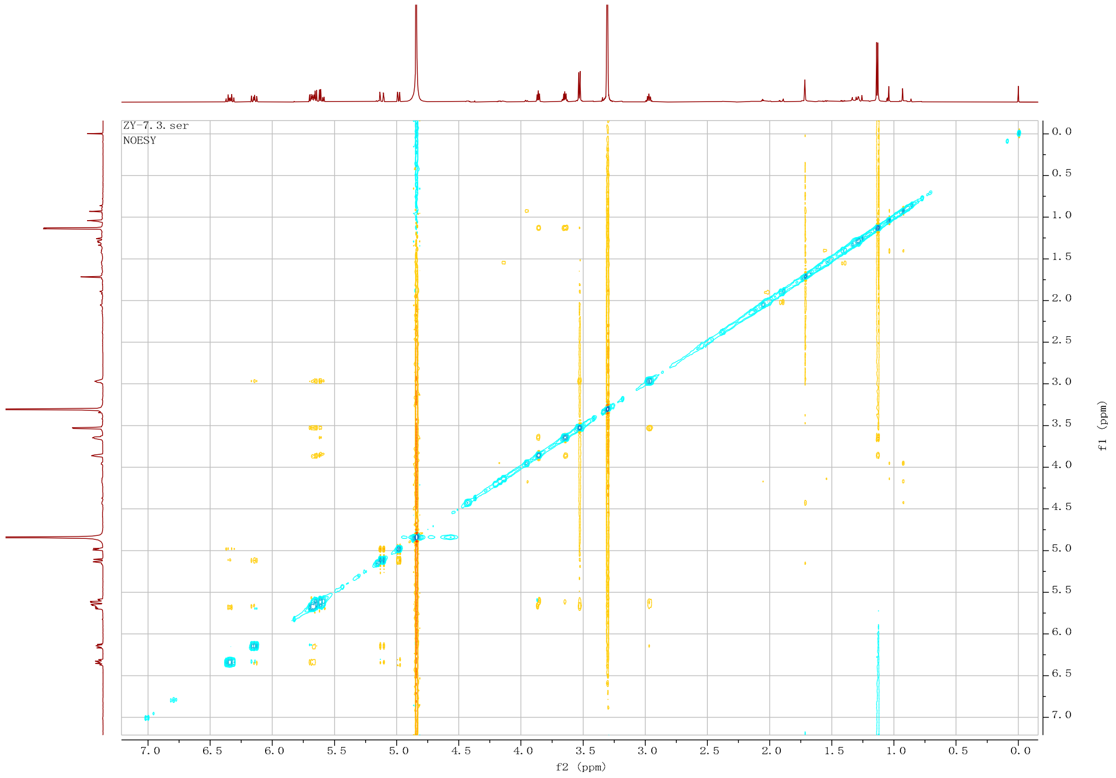

Figure S19. HR-ESI-MS of (5*R*, 9*S*, 10*S*)-5-(hydroxymethyl)-1,3,7-decatriene-9,10-diol (**2**).

Spectrum

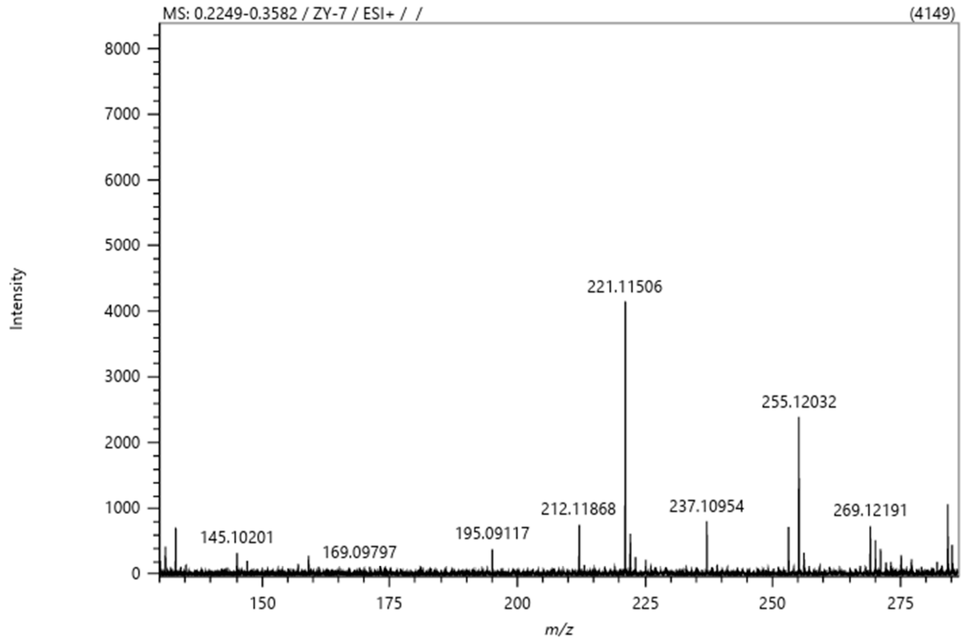

Elemental Composition

Parameters	
Tolerance:	±5.00 ppm
Electron:	Odd/Even
Charge:	+1
DBE:	-1.5 - 200.0

Elements Set 1:

Symbol	C	H	N	O	Na	S	Cl	Br
Min	0	0	0	0	1	0	0	0
Max	200	200	0	8	1	0	0	0

Symbol	Si	F
Min	0	0
Max	0	0

Results

Mass	Intensity	Intensity [%]	Formula	Calculated Mass	Mass Difference [mDa]	Mass Difference [ppm]	DBE
221.11506	4149.26	27.30	C11 H18 O3 Na	221.11482	0.25	1.11	2.5

Figure S20. ORD of (5*R*, 9*S*, 10*S*)-5-(hydroxymethyl)-1,3,7-decatriene-9,10-diol (**2**).

SIOC
Lingling Road 345
Shanghai
China

Anton Paar MCP 5500 - Measurement Results:

Software version: 4.00.11383.92
MCP serial number: 99030100

Sample Information:

- Unique Sample Id: 50936
- Date: 2022-12-16
- Time: 13:30:35
- Method: Specific Rotation (25°C)
- Master Condition: valid
- Sample Name: ZS23
- Concentration: 0.0500 g/100cm³
- User: student

Measurement Result:

Sub Measurement Number	Unique Sample Id	Time	Optical Rotation	Sample Cell Temperature	Specific Rotation (calc.)
			[°]	[°C]	[°]
1	50937	13:29:17	-0.0211	24.88	-42.1958
2	50938	13:29:38	-0.0219	24.94	-43.7956
3	50939	13:29:57	-0.0226	24.98	-45.1955
4	50940	13:30:16	-0.0233	25.01	-46.5953
5	50941	13:30:34	-0.0239	25.04	-47.7952
average	50936	13:30:35	-0.0226	24.97	-45.1155
std. dev.			0.000991	0.0559	1.982109

GxP Information (at 589 nm):

- Last Quartz Adjustment: 2021-11-30 15:41:39 by Administrator

20221216 | 13:30:54

()

Figure S21. ECD of (5*R*, 9*S*, 10*S*)-5-(hydroxymethyl)-1,3,7-decatriene-9,10-diol (**2**) (Six additional isomers)

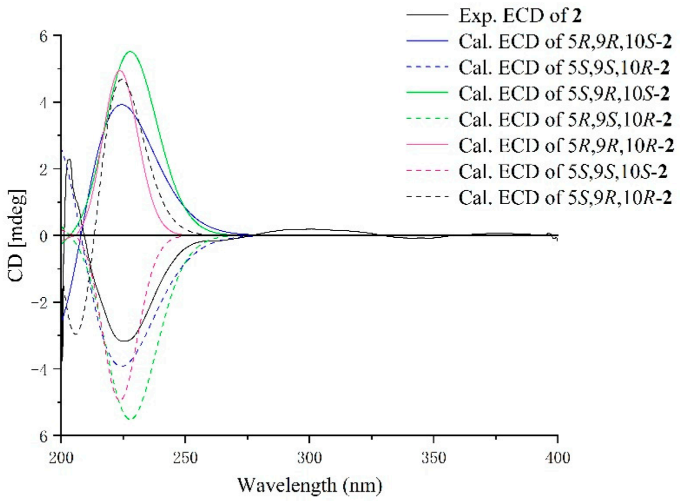

Supplement: Supplementary file 1 [file marinedrugs-22-00331-s001.zip › marinedrugs-3087494-SI.pdf]
